# Different Age-Induced Changes in Rhizosphere Microbial Composition and Function of *Panax ginseng* in Transplantation Mode

**DOI:** 10.3389/fpls.2020.563240

**Published:** 2020-11-12

**Authors:** Qiuxia Wang, Hai Sun, Meijia Li, Chenglu Xu, Yayu Zhang

**Affiliations:** ^1^ Institute of Special Wild Economic Animal and Plant Sciences, Chinese Academy of Agricultural Sciences, Jilin, China; ^2^ College of Pharmacy and Biological Engineering, Chengdu University, Chengdu, China

**Keywords:** transplantation mode, cultivation years, microbial community, microbial functions, rhizosphere soils

## Abstract

Transplantation is a cultivation mode widely applied in perennial plant growing. This method might be an effective way to alleviate problems associated with continuous cultivation (4–6 years) in ginseng production, but the alleviating mechanism and effects on soil microbial community is unclear. To study this issue, non-transplanted 2-year-old, and 5-year-old (transplantation mode: 2 + 3) and 9-year-old (transplantation mode: 3 + 3 + 3) ginseng rhizosphere soils were analyzed *via* MiSeq sequencing. The results showed that 9-year-old ginseng rhizosphere soil had lower available nitrogen and the lowest pH, available phosphorus, observed species and community diversity and richness (Chao1, and ACE) among all samples (*p* < 0.05). The abundances of some bacterial classes (Thermoleophilia, Bacilli, and Nitrospira) and fungal genera (*Mortierella*, *Epicoccum*, and *Penicillium* spp.) and functional richness associated with nutrient element cycles and antifungal activity decreased, while abundances of some fungal genera (*Ilyonectria*, *Tetracladium*, and *Leptodontidium* spp.) increased with increasing age of ginseng plants (*p* < 0.05 or *p* < 0.01). However, there was greater similarity between soil samples of 2-year-old and transplanted 5-year-old ginseng plants and the increase in cultivation time from 2 to 5 years did not significantly influence the microbial community, suggesting that transplantation is a viable strategy for suppressing soil-borne diseases in *Panax ginseng* plants over long growth periods.

## Introduction

Soil sickness causes a reduction in crop yield and a prevalence of soil borne diseases, and it is a negative plant-soil feedback with the same crop growing on the same soil successively (Huang et al., 2013). Soil sickness is a common phenomenon not only for many crops such as maize (Gentry et al., 2013), peanut (Li et al., 2018a), cucumber (Jin et al., 2019), but also for many perennial medicinal plants (Xiao et al., 2016a; Tan et al., 2017a). For example, *Panax ginseng* (Araliaceae family) is a perennial herbaceous plant that is widely cultivated in northeast China, Korea, and Japan for its highly valued root, which possesses multifunctional properties (Yang et al., 2014; Shin et al., 2015; Xu et al., 2017). Moreover, the potency of pharmacological/bioactive constituents in ginseng root tends to increase with cultivation age (Shi et al., 2007; Li et al., 2014a). Generally, it takes at least 5–6 years before ginseng is ready to be harvested (Ying et al., 2012). However, the risks for soil-borne diseases increase over longer periods of cultivation, and these factors have severe negative effects on the yield and quality of ginseng (Ying et al., 2012; Li et al., 2014b).

The causal agents of soil sickness have also been proved to be a combination of biotic and abiotic factors (Huang et al., 2013). Regarding the biotic factors, rhizosphere microorganisms are considered to be the important indicators of soil function that significantly affect the growth, nutrition, and health of plants (Franke-Whittle et al., 2015; López-Carmona et al., 2019). An imbalance in these communities is responsible for the soil sickness, as the abundances of some microorganisms decrease, including *Pseudomonas*, *Bacillus*, and arbuscular mycorrhizal fungal species, which can prevent plant disease and improve growth (Li et al., 2012, 2014b; Kil et al., 2014). In contrast, pathogenic fungi such as *Cylindrocarpon*/*Ilyonectria*, unclassified genera *Leotiomycetes*, and *Fusarium*, which are associated with soil-borne diseases, tend to pose greater risks to plants with increasingly long growth periods of 4–6 years (Xiao et al., 2016a; Tan et al., 2017a; Dong et al., 2018). In addition, abiotic factors such as the soil pH and fertility play vital roles in ginseng growth, which requires slightly acidic and nutrient-rich soil. The soil pH and fertility have been shown to be strongly linked with soil microbial communities (Shen et al., 2013; Siciliano et al., 2014). In the rhizosphere of ginseng plants, the soil pH and nutrient concentrations decline with increasing years of cultivation, ultimately leading to decreased microbial diversity, which in turn is responsible for the development of soil-borne ginseng diseases (Nguyen et al., 2016a; Xiao et al., 2016a; Dong et al., 2017). It has also been demonstrated that allelochemicals (root exudates) of *P. ginseng* significantly decrease the genetic diversity and carbon-metabolic activity of microorganisms and cause chemotaxis responses of ginseng pathogenic microorganisms (Li et al., 2014c, 2016; Lei et al., 2017). Consequently, negative plant-soil feedbacks *via* pathogenic activity, deteriorated soil conditions, or allelopathy, played an important role in soil sickness (Huang et al., 2013; Zhou et al., 2018).

A transplantation mode involves transplantation of ginseng to a new location after growing in one place for 2–3 years (Li et al., 2014c). It is an effective way to avoid the excessive accumulation of allelochemicals and soil deterioration that occurs when the same plants are cultivated in one field for years. This practice is common in the cultivation of the perennial herbaceous plant widely used in traditional Chinese medicines for their highly valued root (Xiao et al., 2016a). For example, 5-year-old ginseng in transplantation mode requires direct sowing and growing in one field for 2–3 years; it is then transplanted to a new field with no recent ginseng plantation history, where it grows for another 2–3 years. Many studies have proved that microbial community became unbalanced, and phytopathogens would gradually be the predominant in the rhizosphere soil of *P. ginseng* (Li et al., 2012; Ying et al., 2012). In addition, ginseng soil samples without transplantation have lower microbial diversity than ginseng soil samples with transplantation (Nguyen et al., 2016a; Xiao et al., 2016a). Because of the importance of transplantation mode, it is necessary to explore the changes in soil abiotic factors and microbial communities associated with transplantation mode. This study will increase our understanding of the status of rhizosphere microbial communities in relation to years of the perennial herbaceous plant growing in transplantation mode and will help in field management with respect to perennial plant cultivation. However, the composition and function of rhizosphere microbial community following different numbers of years in transplantation mode remain poorly understood and is due to complicated metabolic pathways of microbial community and methodological limitations (Xiao et al., 2016a; Wang et al., 2018). Amplicon-based studies involving 16S rRNA genes or internal transcribed spacer (ITS) sequences have been an effective way to achieve high sample-throughput and a deeper insight into soil microbial communities (Miao et al., 2016; Sun et al., 2017; Xiong et al., 2017). Recently, FAPROTAX and FUNGuild were developed to predict the functions, lifestyles, or guilds of bacterial and fungal communities following the data from high-throughput sequencing, respectively (Louca et al., 2016; Nguyen et al., 2016b). These two bioinformatic tools were employed to analyze bacterial functional diversity and fungal trophic mode in soil (Bao et al., 2018; Tayyab et al., 2019); thus, they can provide the potential ways to decipher function succession of rhizosphere microbial community associated with the transplantation mode.

In our study, rhizosphere microbial composition and functional potential of *P. ginseng* at various ages in transplantation mode were investigated by MiSeq sequencing of the 16S rRNA gene and ITS1 region, and FAPROTAX and FUNGuild tools. The objectives of the present study were (1) to characterize the rhizosphere microbial community composition and functional profiling of *P. ginseng* at various ages in transplantation mode, and (2) to assess the relationships between soil properties and the rhizosphere microbial communities of *P. ginseng*. We demonstrated that (1) microbial community diversity decreased, and the soil microbial community composition and function changed with increasing number of years of cultivation, especially with 9-year-old transplanted ginseng plants and (2) there would not be significant difference in microbial communities between 2-year-old and 5-year-old ginseng rhizosphere soils in transplantation mode. This study will increase our understanding of the status of rhizosphere microbial communities in relation to years of the perennial herbaceous plant growing in transplantation mode and will help in soil amelioration after ginseng cultivation.

## Materials and Methods

### Soil Sampling

The main production region of *P. ginseng* is Fusong in the Changbai Mountains of China. A mixture of local soils, humus and albic horizons (1:1), one of the main soil types in Fusong, were used to create raised ginseng bed soils for ginseng cultivation (You et al., 2015; Wang et al., 2019a). In September 2014, soil samples of *P. ginseng* cultivated in transplantation mode for increasing times were obtained from five ginseng fields (named A–E) from three locations (Manjiang, Donggang, and Wanliang Town) in Fusong (Table 1; Wang et al., 2016). Because ginseng is usually harvested at the age of 5 years, soil samples of 5-year old ginseng plants from three fields (A, C, and D; Table 1) in transplantation mode were selected. Disease occurrence and mortality rates in ginseng seedlings generally increase after 2 years of consecutive growth (Dong et al., 2018); therefore, soil samples from non-transplanted 2-year-old ginseng plants were chosen as the control in this study (soil E in Table 1). Soil samples from ginseng plants in transplantation mode with a high age (9 years old) were also studied (soil B in Table 1). G2, G5, and G9 represent soil samples from 2-year-old, 5-year-old, and 9-year-old ginseng plants, respectively, which were obtained as follows:

1. G2 ginseng plants: direct sowing and growing for 2 years without transplantation;
2. G5 ginseng plants: direct sowing and growing in one field for 2 years with subsequent transplantation to a second field, where they grew for 3 years before sample collection;
3. G9 ginseng plants: direct sowing and growing in one field for 3 years, followed by transplantation to another field for 3 years, and final transplantation to a third field, where they grew for another 3 years before sampling.

**Table 1:** Soil samples used in the present work.

Fields	Transplantation mode^a^	Start time	Transplanting time	Harvesting time	Cultivation years^b^	Location
A	2 + 3	2009	2011	2014	5 (G5)	Manjiang Town, Fusong
B	3 + 3 + 3	2005	2008, 2011	2014	9 (G9)	Manjiang Town, Fusong
C	2 + 3	2009	2011	2014	5 (G5)	Donggang Town, Fusong
D	2 + 3	2009	2011	2014	5 (G5)	Wanliang Town, Fusong
E	2	2012	/	2014	2 (G2)	Wanliang Town, Fusong

a In the transplantation mode column, the number presented (i.e., 2 + 3) indicates that *Panax ginseng* plants were grown in one field for 2 years and then transplanted to the indicated sampling field and grown for 3 years. Similarly, 3 + 3 + 3 indicates cases, where plants were directly seeded and grown in one field for 3 years, then transplanted to another field for 3 years, and finally transplanted to the indicated sampling field and grown for 3 years.

b G2, G5, and G9 indicate soil samples from 2-year, 5-year, and 9-year-old ginseng plants, respectively.

In our study, ginseng seeds were sowed and all transplanted plants were replanted in soils with no recent history of ginseng cultivation. Forty to sixty rhizosphere soil samples were randomly collected by gently scraping the soil directly attached to the ginseng roots from each ginseng field. Ten soil samples from 10 ginseng roots with the same rust area were uniformly mixed after removal of visible plant materials and were considered one replicate (Wang et al., 2019a). All soil samples were placed on ice for 1–2 days, after which they were passed through a 2-mm sieve and homogenized. Soil samples were stored at −80°C until DNA extraction. For soil properties, soil samples were air-dried and passed through a 0.15 mm sieve for organic matter (OM) content analysis, and a 2 mm sieve for pH, available nitrogen (AN), available phosphorus (AP), and available potassium (AK) content, respectively. Soil properties, including the pH, OM, AN, AP, and AK, were determined as previously described (Wang et al., 2016).

### Soil DNA Extraction, PCR Amplification, and Illumina Miseq Sequencing

Total DNA from 0.50 g of each soil sample was extracted using the PowerSoil® DNA Isolation Kit (MoBio Laboratories, CA, USA) according to the manufacturer’s instructions and was quantified using a NanoDrop2000 device (Thermo Scientific, Pittsburgh, PA, USA). Each soil sample was extracted in triplicate, and the three DNA solutions were combined together.

DNA was amplified by the PCR using the primer set 341F (5'-CCTACGGGNGGCWGCAG-3') and 805R (5'-barcode-GACTACHVGGGTATCTAATCC-3') for the V3–V4 regions of 16S rRNAs, or ITS1F (5'-CTTGGTCATTTAGAGGAAGTAA-3') and ITS1R (5'-barcode-ATGAGCGCTGCGTTCTTCATCGATGC-3') for the fungal ITS1 sequences. Sample-specific barcodes were incorporated into the primers. The PCR conditions used were as follows: 95°C for 2 min; 27 cycles of 95°C for 30 s, 55°C for 30 s, and 72°C for 45 s; final extension at 72°C for 10 min; and a hold at 10°C. Only one PCR was performed for each soil sample with 20-μl reaction mixtures containing 4 μl of 5× FastPfu Buffer, 2 μl of 2.5 mM dNTPs, 0.4 μl of each primer (5 μM), 0.4 μl of TransStart FastPfu DNA Polymerase (TransGen Biotech, Beijing, China), and 10 ng of template DNA. Amplicons were extracted from 2% agarose gels and purified using the AxyPrep DNA Gel Extraction Kit (Axygen Biosciences, Union City, CA, USA), following the manufacturer’s instructions and then quantified using QuantiFluor™-ST (Promega, Madison, WI, USA). The normalized PCR products were subjected to paired-end sequencing using MiSeq platform (Illumina, San Diego, CA, USA) at SinoGenoMax (Beijing) according to the standard protocol.

### Sequence Data Processing

After removing the barcode and primer sequences using MOTHUR (Schloss et al., 2009), the remaining reads were merged and quality filtered using FLASH (Magoč and Salzberg, 2011) and QIIME (Caporaso et al., 2010). Following the removal of chimeric sequences by UCHIME (Edgar et al., 2011), operational taxonomic units (OTUs) were clustered at 97% sequence similarity using the UPARSE pipeline (Edgar, 2013). Finally, the taxonomic affiliation of each OTU was calculated using the RDP classifier (version 2.2; Wang et al., 2007) against the Silva128 Database for bacteria (confidence coefficient = 0.8–1) and the UNITE_INSD v7.0 Fungal ITS database for fungi (E value = 1e–05; Kõljalg et al., 2013). The taxon abundances in each sample were generated at the phylum, class, order, family, and genus levels. Sequences were deposited in the NCBI Short Read Archive under accession numbers SRP131809 and SRP129584.

### Statistical Analysis

After removing singletons, the alpha diversity was calculated with QIIME (version 1.7.0) based on the Shannon, Simpson, Chao1, and ACE diversity indices. Statistical analysis was carried out using the SAS 9.1 software package (SAS institute Inc., Cary, NC, USA). One-way ANOVA with least significant difference (LSD) test was used to compare the means of samples with 4–6 replicates, and variability in the data was expressed as the standard error. Differences at *p* < 0.05 and *p* < 0.01 were considered significant and highly significant, respectively.

Microbial community similarities among the different samples were determined by performing UniFrac analyses (Lozupone et al., 2011). Principal coordinates analysis (PCoA) based on the weighted UniFrac distance and analysis of similarities (ANOSIM) were used to depict differences in the microbial community compositions. A heatmap was generated using the gplots package in R (version 2.15.3) to compare the top 35 bacterial classes and fungal genera in soil samples with different years of cultivation. Redundancy analysis (RDA) has been performed to measure the linkage between variables of soil microbial community and soil factors by CANOCO5.0 (Biometrics Wageningen, the Netherlands; Xiao et al., 2016b). Spearman’s correlation analyses were also performed to assess the relationships among the soil properties, plant age, and microbial community, using the Vegan package of R software (Oksanen et al., 2019). The functions, lifestyles, or guilds of the bacterial and fungal OTUs were identified using FAPROTAX (Louca et al., 2016) and FUNGuild (Nguyen et al., 2016b), respectively. Differences in functional groups between different soils were determined using ANOVA.

## Results

### Rhizosphere Soil Properties of *P. ginseng*

The rhizosphere soil properties (pH, AN, AP, AK, and OM) were strongly influenced by the plant age (Table 2). Compared with the G2 soil samples, ANOVA showed that the pH and AN were significantly lower in G5 by 13.90 and 30.32%, respectively, and in G9 by 27.32 and 48.21%, respectively, (*p* < 0.05), whereas the OM was markedly higher in G5 by 249.87% and in G9 by 181.87% (*p* < 0.05), respectively. The level of AP did not notably differ between G2 and G5 soils. However, the AP levels of G9 were notably lower than those in G2 by 61.32%, and G5 by 72.74% (*p* < 0.05), respectively. Furthermore, no significant difference was found in AK across all soil samples with different transplantation treatments.

**Table 2:** The basic properties of soil samples used in the present work.

Fields	Cultivation years	pH	OM (g/kg)	AN (mg/kg)	AP (mg/kg)	AK (mg/kg)
E	2 (G2)	6.26 ± 0.18^a^	34.37 ± 2.40^b^	565.84 ± 41.32^a^	32.73 ± 8.16^a^	325.00 ± 78.01^a^
A, C, and D	5 (G5)	5.39 ± 0.07^b^	120.25 ± 7.95^a^	394.25 ± 30.14^b^	46.44 ± 3.54^a^	252.72 ± 18.80^a^
B	9 (G9)	4.55 ± 0.08^c^	96.88 ± 7.98^a^	293.05 ± 26.11^c^	12.66 ± 1.50^b^	327.75 ± 27.61^a^

The OM, AN, AP, and AK represented, respectively, the contents of organic matter, available nitrogen, available phosphorus, and available potassium; means followed by different letters (a-c) within a column are significantly different as determined by the LSD test (*p* < 0.05).

### Microbial Diversity and Structure

The alpha-diversity indices, including the number of observed species and diversity and richness indices of the 16S rRNA bacterial and ITS fungal libraries, were different across all soil samples. For bacteria, G9 soil (B) had the lowest number of observed species (882) and community diversity and richness (Shannon = 8.451, Simpson = 0.994, Chao1 = 1,271, and ACE = 1,325) among all samples (*p* < 0.05); for fungi, the lowest observed species number (705) and richness (Chao1 = 997 and ACE = 1,022) were also observed in G9 (B; *p* < 0.05; Table 3). However, no significant differences in any of the bacterial and fungal alpha-diversity indices were found between the G2 and G5 groups.

**Table 3:** Number of observed species, and diversity and richness indices of the 16S rRNA bacterial and ITS fungal libraries obtained by clustering at 97% identity.

	Cultivation years	Observed species	Shannon	Simpson	Chao1	ACE
	2 (G2)	1,220 ± 112^a^	9.351 ± 0.181^a^	0.997 ± 0.0002^a^	1,583 ± 186^a,b^	1,676 ± 207^a^
Bacteria	5 (G5)	1,300 ± 24^a^	9.381 ± 0.053^a^	0.997 ± 0.0002^a^	1866 ± 43^a^	1973 ± 43^a^
	9 (G9)	882 ± 67^b^	8.451 ± 0.175^b^	0.994 ± 0.0009^b^	1,271 ± 145^b^	1,325 ± 129^b^
	2 (G2)	883 ± 83^a^	6.645 ± 0.526^a^	0.949 ± 0.022^a^	1,344 ± 198^a^	1,207 ± 80^a^
Fungi	5 (G5)	829 ± 23^a^	6.598 ± 0.127^a^	0.966 ± 0.005^a^	1,119 ± 44^a,b^	1,171 ± 41^a^
	9 (G9)	705 ± 41^b^	6.194 ± 0.240^a^	0.954 ± 0.010^a^	997 ± 52^b^	1,022 ± 50^a^

Means followed by the different letters (a,b) within a column represent significant differences, as determined by the LSD test (*p* < 0.05).

**Figure 1:**
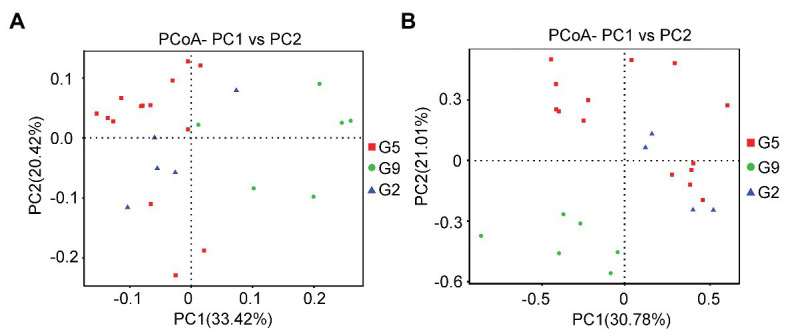
Different analyses of the microbial community structures based on operational taxonomic units (OTUs). **(A)** Principal coordinates analysis (PCoA) graph based on the 16S rDNA sequence. **(B)** PCoA graph based on the ITS1 sequence.

The beta diversities of the soil microbial communities in *P. ginseng* rhizosphere soil at varying ages were evaluated using PCoA (Figure 1) and ANOSIM analysis (Table 4). Differences in the microbial communities were observed, especially between the G9 group and the other two groups (G2 and G5); however, most of the soil microbial communities in the G2 and G5 samples clustered together (Figure 1). In the pairwise ANOSIM test, the lowest *R* values were observed between G2 and G5 (*R* ≤ 0.300, *p* < 0.05), whereas a much higher R value was observed between G9 and the other two samples (G2 and G5; *R* ≥ 0.626, *p* < 0.05) for both bacteria and fungi (Table 4). Thus, the PCoA analysis and ANOSIM test suggested that the closest similarity existed between the non-transplanted G2 samples and the transplanted G5 samples.

**Table 4:** Dissimilarities in the microbial community composition across different groups as determined by analysis of similarities (ANOSIM).

Cultivation years	Bacteria		Fungi	
	*R*	*p*	*R*	*p*
G2–G5	0.278	0.046	0.300	0.047
G5–G9	0.834	0.001	0.626	0.001
G2–G9	0.888	0.007	0.980	0.011

An *R*-value near +1 indicates that dissimilarity was observed between the groups, whereas an *R*-value near 0 indicates that no significant dissimilarity was observed between the groups. *p* < 0.05 indicates significant dissimilarity; G2, G5, and G9 indicate soil samples from 2-year, 5-year, and 9-year-old ginseng plants, respectively.

### Bacterial Community Composition and Function Analysis

**Figure 2:**
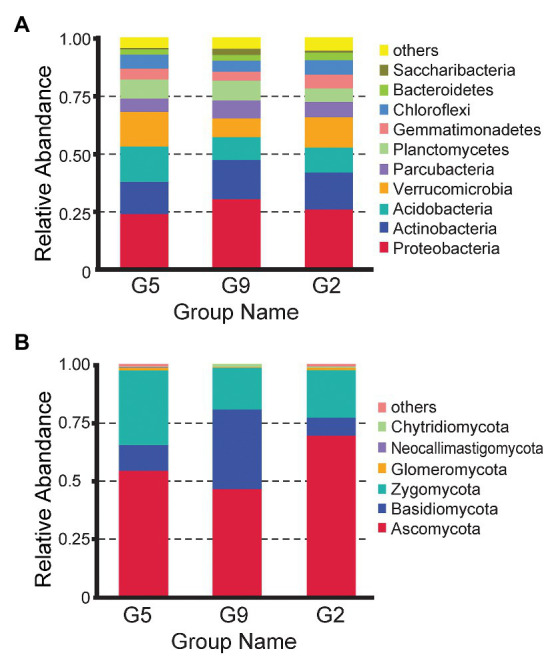
Comparison of the bacterial **(A)** and fungal **(B)** communities from G2, G5, and G9 soil samples at the phylum level.

The effective bacterial sequences were all assigned to 39 phyla using QIIME with the default settings, and the three predominant phyla across all samples were Proteobacteria (25.59–32.38%), Actinobacteria (12.56–15.20%), and Acidobacteria (11.15–16.51%) across the samples with different years (Figure 2A). Different cultivation years significantly changed the relative abundances of the major bacterial phyla. The relative abundances of Proteobacteria (32.38%) and Saccharibacteria (3.44%) phyla were markedly higher in the rhizospheres of older ginseng plants (G9), whereas Nitrospirae (0.97 and 1.14%), Chloroflexi (6.94 and 6.54%), and Elusimicrobia (0.20 and 0.16%) phyla were higher in those of younger ginseng plants (G2 soil and G5 soils; *p* < 0.05). The Bacteroidetes phylum (3.82%) showed the highest abundance in the G2 samples (*p* < 0.05).

**Figure 3:**
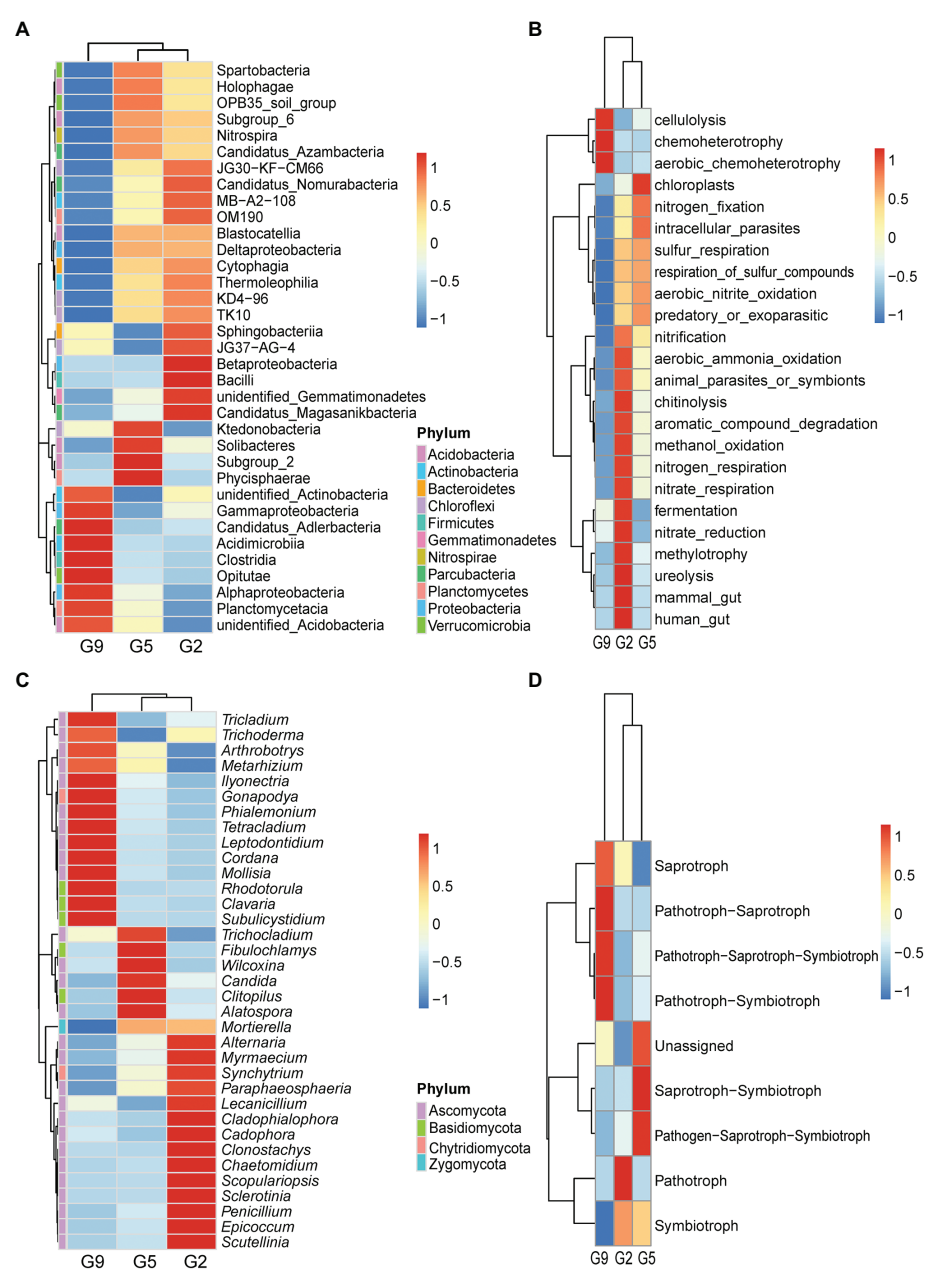
Variations in microbial community compositions and functional characteristics in G2, G5, and G9 soil samples. Heatmap analyses of the abundances of the top 35 bacterial classes **(A)** and fungal genera **(C)**. Functional analysis was performed using FAPROTAX for bacteria **(B)** and FUNGuild for fungi **(D)**, respectively.

Furthermore, a comparison of the top 35 classes from the predominant phyla revealed that the G2 and G5 samples clustered together and had similar microbial community structures at the class level with most classes being found in soils samples from both groups. However, only nine predominant classes were found in the G9 soil samples. Specifically, Thermoleophilia (4.25 and 3.97%), Deltaproteobacteria (2.54 and 2.58%), TK10 (0.46 and 0.43%), KD4.96 (0.82 and 0.68%), and Nitrospira (1.13 and 1.30%) classes were more abundant, respectively, in G2 and G5 samples, whereas Gammaproteobacteria (7.66%) and Alphaproteobacteria (19.05%) classes were more abundant in G9 samples (*p* < 0.05 or *p* < 0.01). Betaproteobacteria (6.17%) and Bacilli (1.16%) classes were only detected in high abundance in the G2 group (*p* < 0.05; Figure 3A). Differences in bacterial functions between the G2, G5, and G9 groups were investigated using FAPROTAX (Figure 3B). G9 had the lowest functional richness in terms of only three functions. Long cultivation years (>5 years) significantly increased functional groups of cellulolysis (5.79%), chemoheterotrophy (15.50%), and aerobic chemoheterotrophy (14.97%; *p* < 0.05 or *p* < 0.01), and decreased functional groups of aerobic ammonia oxidation (0.39%), nitrification (0.57%), respiration of sulfur compounds (0.05%), sulfur respiration (0.04%), nitrogen fixation (0.48%), and aerobic nitrite oxidation (0.18%; *p* < 0.05 or *p* < 0.01). Some functional groups associated with nutrient element cycles (e.g., methylotrophy, methanol_oxidation nitrification, respiration of sulfur compounds, sulfur respiration, nitrogen fixation, and aerobic nitrite oxidation) and antifungal activity (chitinolysis) were markedly enriched in G2 or G5, which were 1.48–19.37 times higher than that in G9 (*p* < 0.05 or *p* < 0.01). G2 had the highest functional richness in terms of 14 functions.

**Figure 4:**
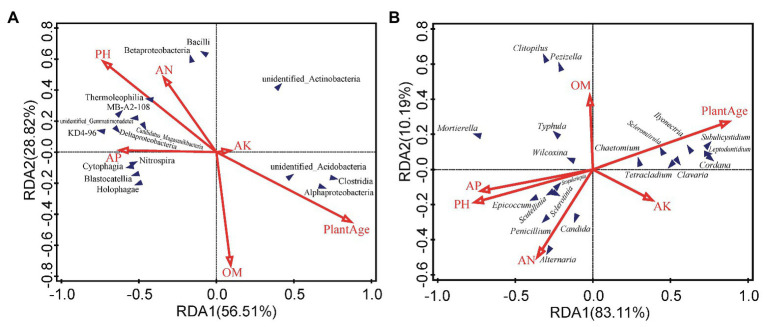
Redundancy analysis (RDA) to measure the linkage between variables of soil microbial community and soil factors, based on the relative abundance of bacterial classes **(A)** and fungal genera **(B)**. OM, organic matter; AN, available nitrogen; AP, available phosphorous; and AK, available potassium.

**Figure 5:**
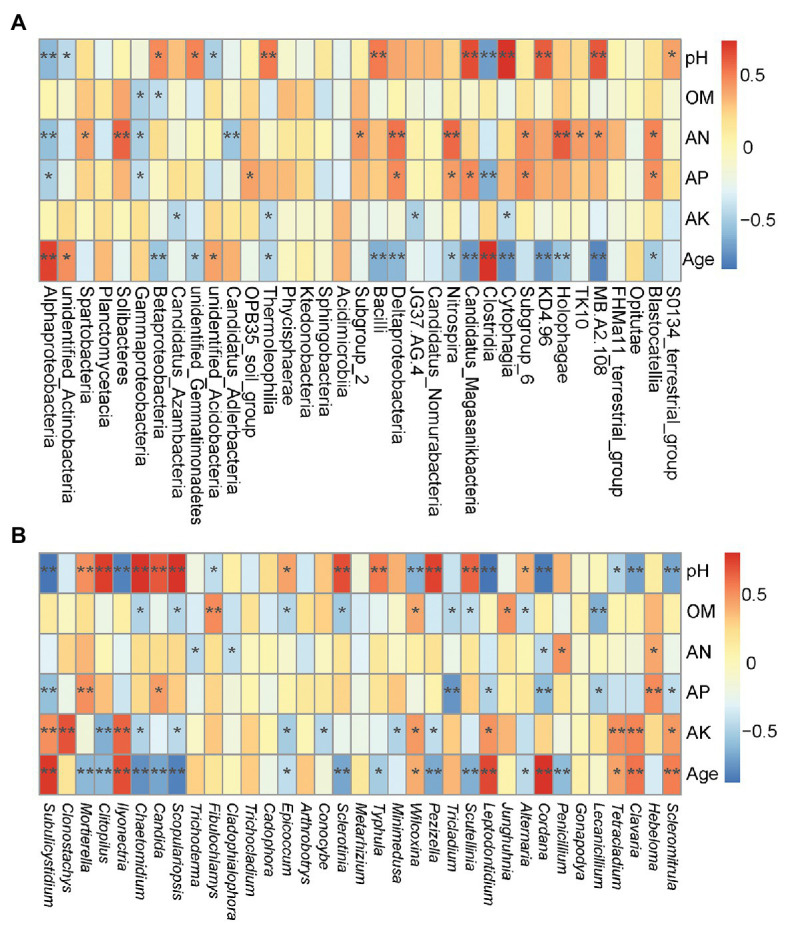
Spearman’s correlation analyses between the soil properties and plant age, and abundance of the top 35 bacterial classes **(A)** and fungal genera **(B)**. ^**^ *p* < 0.01; ^*^ *p* < 0.05. OM, organic matter; AN, available nitrogen; AP, available phosphorous; and AK, available potassium.

Environmental factors, such as pH, AN, AP, OM and plant age appear to be the most important environmental factors (Figure 4A). According to the RDA analysis, Deltaproteobacteria, Betaproteobacteria, Thermoleophilia, Bacilli, and Nitrospira classes were positively affected by pH, AN, and AP, and negatively affected by plant age, and even Betaproteobacteria, Thermoleophilia, and Bacilli were negatively affected by OM, whereas Alphaproteobacteria, and unidentified_Acidobacteria classes were negatively affected by pH, AN, and AP, and positively affected by plant age. Moreover, Spearman’s rank correlations revealed that approximately 12 classes, including Thermoleophilia, Bacilli, and Nitrospira, showed significant negative correlations with the plant age (*r* = −0.44, *p* < 0.05; *r* = −0.61, *p* < 0.01; *r* = −0.47, *p* < 0.05), or Thermoleophilia negatively correlated with AK (*r* = −0.42, *p* < 0.05), but Thermoleophilia and Bacilli were positively correlated with the pH(*r* = 0.54, *p* < 0.01and *r* = 0.53, *p* < 0.01), or Nitrospira was positively correlated with AP (*r* = 0.42, *p* < 0.05) and AN(*r* = 0.58, *p* < 0.01). In contrast, three classes (Alphaproteobacteria, unidentified_Actinobacteria, and unidentified_Acidobacteria) were positively correlated with the plant age (*r* = 0.70, *p* < 0.01; *r* = 0.47, *p* < 0.05; *r* = 0.40, *p* < 0.05), but negatively with the pH(*r* = −0.58, *p* < 0.01; *r* = −0.42, *p* < 0.05; and *r* = −0.48, *p* < 0.05), or Alphaproteobacteria negatively with AP (*r* = −0.47, *p* < 0.05) and AN (*r* = −0.55, *p* < 0.01; Figure 5A). At the OTU levels, six OTUs (OTU11, OTU13, OTU15, OTU29, OTU45 and OTU46) belonging to Alphaproteobacteria and Gammaproteobacteria classes were also more abundant in G9 samples (1.38, 1.14, 1.22, 1.30, 0.92, and 0.58%; *p* < 0.05 or *p* < 0.01); and two OTUs (OTU663 and OTU1212) belonging to Bacilli class were also only detected in high abundance in the G2 group (0.32 and 0.28%; *p* < 0.05).

### Fungal Community Composition and Function Analysis

Fungal sequences were only assigned to six phyla according to QIIME using default settings. Fungal sequences were predominantly associated with the phyla Ascomycota (47.04–70.25%), Zygomycota (17.93–32.18%), and Basidiomycota (7.67–34.40%), whereas Glomeromycota (or Glomeromycotina; 0.34–1.07%), Chytridiomycota (0.27–0.57%), and Neocallimastigomycota (0.02–0.23%) were present in most soils, but at relatively low abundance (Figure 2B). The abundance of some phyla varied in different soil samples. For example, Ascomycota and Zygomycota were most prevalent in G2 (70.25 and 20.41%, respectively) and G5 (54.95 and 32.18%, respectively) but had low abundances in G9 (47.04 and 17.93%, respectively; *p* < 0.05). In contrast, Basidiomycota was most abundant in G9 (34.40%; *p* < 0.05).

At the genus level, a heatmap analysis of the top 35 fungal genera also revealed significant differences in the fungal community compositions of ginseng soils at various ages (Figure 3C). G2 was enriched with *Penicillium* and *Epicoccum* spp., which respectively were 4.22–7.94 and 4.69–7.16 times higher than in G5 and G9 soils (*p* < 0.05). G2 and G5 were enriched with *Mortierella* spp. 2.59–2.65 higher than in G9 soil. G9 showed 3.04–6.52, 2.50–3.02, and 9.81–27.90 times higher abundances of *Ilyonectria*, *Tetracladium*, and *Leptodontidium* spp. than in G2 and G5, respectively (*p* < 0.05). There were seven major fungal trophic modes detected in all samples (Figure 3D). These modes were varied among different samples, and most pathotrophic relationships (pathotroph-saprotroph, pathotroph-saprotroph-symbiotroph, and pathotroph-symbiotroph) were enriched in G9 soil, which were 4.13–4.19, 2.12–3.21, 2.26–3.17 times higher than in G2 and G5 soils, respectively (*p* < 0.05).

According to the RDA analysis, *Epicoccum*, *Mortierella* and *Penicillium* spp. were negatively affected by plant age, positively affected by pH and AP, and even *Epicoccum* and *Penicillium* spp. were positively affected by AN, whereas *Ilyonectria*, *Tetracladium*, and *Leptodontidium* spp. were negatively affected by pH, AN, and AP, and positively affected by plant age (Figure 4B). Furthermore, Spearman’s rank correlations were also employed to evaluate the relationships between soil properties and the top 35 fungal genera (Figure 5B). Twelve genera, including *Mortierella*, *Epicoccum*, and *Penicillium* spp., were negatively correlated with the plant age (*r* = −0.59, *p* < 0.01; *r* = −0.42, *p* < 0.05; and *r* = −0.57, *p* < 0.01), and even *Epicoccum* spp. were negatively correlated with OM (*r* = −0.44, *p* < 0.05) and AK (*r* = −0.50, *p* < 0.05). However, *Mortierella* and *Epicoccum* spp., etc. displayed a significant positive correlation with the pH (*r* = 0.52, *p* < 0.01 and *r* = 0.46, *p* < 0.05), and *Mortierella* and *Penicillium* spp., were positively correlated with AP (*r* = 0.52, *p* < 0.01) or AN (*r* = 0.51, *p* < 0.05), respectively. In contrast, eight genera, including *Ilyonectria*, *Leptodontidium*, *Tetracladium*, and *Scleromitrula* spp., were positively correlated with the plant age (*r* = 0.72, *p* < 0.01; *r* = 0.75, *p* < 0.01; *r* = 0.47, *p* < 0.05 and *r* = 0.58, *p* < 0.01) and AK (*r* = 0.66, *p* < 0.01; *r* = 0.50, *p* < 0.05; *r* = 0.53, *p* < 0.01 and *r* = 0.49, *p* < 0.05) but were negatively correlated with the pH (*r* = −0.79, *p* < 0.01; *r* = −0.86, *p* < 0.01; *r* = −0.46, *p* < 0.05 and *r* = −0.66, *p* < 0.01). In addition, *Leptodontidium* and *Scleromitrula* spp. were negatively correlated with AP (*r* = −0.47, *p* < 0.05 and *r* = −0.41, *p* < 0.05). At the OTU levels, two OTUs (OTU1 and OTU4) belonging to *Mortierella* spp. were also more abundant in G2 (8.24 and 5.16%) and G5 (8.77 and 4.90%) samples (*p* < 0.05).

## Discussion

### Rhizosphere Soil Characteristic Response to Cultivation Years in Transplantation Mode

Soil pH is the primary factor involved in ginseng growth and development. It has been found in previous studies that the soils with ginseng cultivation had a much lower pH values than those without ginseng (You et al., 2015; Xiao et al., 2016a). The soil pH in transplantation mode also decreased with increasing cultivation years in our study (Table 2).

The soil pH can influence the environmental AN, AP, and OM contents by controlling the transformation of N, P, and OM (Šantrůčková et al., 2004; Yao et al., 2011; Sommer et al., 2017). It has been demonstrated that changes in the soil pH significantly affect the rate of soil N cycling, and the absence of nitrification (a crucial N cycling process) in some highly acidic soils resulted in low concentrations of AN (nitrate; De Boer and Kowalchuk, 2001; Kemmitt et al., 2006). Nguyen et al. (2016a) suggested that long-term ginseng cultivation may decrease the soil pH and that the acidic soil can cause P absorption *via* Fe–Al and Ca^2+^ interactions, leading to low soil P concentrations. Owing to the lowest pH, the concentrations of AN and AP deceased in 9-year old ginseng soil (Table 2). Previously, it has been shown that an increased pH drove OM solubilization and transformation, and lowered the soil OM contents (Grybos et al., 2009); conversely, our current findings showed that OM accumulation in soils with older ginseng plants was attributable to a lower soil pH, which prevented OM transformation.

### Rhizosphere Bacterial Community Composition and Functions in Transplantation Mode

The increase of cultivation years (>5 years) had a negative effect on microbial diversity (Table 3). This effect has also been reported previously (Vendan et al., 2012; Li et al., 2014b; Xiao et al., 2016a). However, the lack of differences in microbial diversity between the non-transplanted G2 samples and the transplanted G5 samples suggested that increased cultivation years (≤5 years) did not significantly influence the bacterial diversity of ginseng soil because of transplantation.

Growing ginseng plants for increasing years negatively affected the bacterial composition. Proteobacteria was the major bacterial phylum associated with *P. ginseng* soils (Xiao et al., 2016a; Sun et al., 2017), and different classes of Proteobacteria were found to be associated with *P. ginseng* plants of different ages (Figure 3). The abundances of some bacterial classes (including members of Thermoleophilia, Bacilli, and Nitrospira) that negatively correlated with the plant age (Figures 4A, 5A) proved to be beneficial for plant growth (Kumar et al., 2012; Guan et al., 2013; Zhou et al., 2015). However, some of them (Thermoleophilia and Nitrospira) were enriched in the soil of ginseng plants with lower cultivation years (up to 5 years) in transplantation mode.

Bacterial community composition is very closely related to microbial function. Nitrospirae and Chloroflexi classes have been shown to play key roles in nitration-anammox reactors (Shu et al., 2016; Gu et al., 2019). Some families of Desulfobacterales in Deltaproteobacteria class are closely associated with sulfur (S) cycling (Zhang et al., 2018). In addition, many species of *Bacillus* in Bacilli class can reduce the incidence of soil-borne disease by producing chitinases and acting as biocontrol agents (Kishore and Pande, 2007; Li et al., 2018b). The reduction in bacterial diversity and the abundance of some microbes resulted in a decrease of functional groups involved in nutrient element cycling and soil resistance with higher cultivation years (9-year-old ginseng plants) In contrast, in our study, the functional groups of N and S cycling were higher and might be contributing to the enrichment of Nitrospira, TK10, KD4.96 and Deltaproteobacteria classes in soil of ginseng with lower cultivation years (up to 5 years) in transplantation mode.

### Rhizosphere Fungal Community Composition and Functions in Transplantation Mode

Our result suggested that increasing cultivation years decreased fungal diversity. The decreased fungal diversity could lead to plant disease and increased death rate, whereas increased soil fungal diversity might involve the amelioration of soil sickness (Dong et al., 2016; Tan et al., 2017b). The fungal community compositions of ginseng rhizosphere soil changed with increasing cultivation years, which has also been found in previous study (Dong et al., 2018). The *Mortierella*, *Epicoccum*, and *Penicillium* spp., which were beneficial for plant growth, were negatively correlated with the plant age (Figures 4B, 5B). It was inferred that *Mortierella* spp., which can produce antibiotics and potential antagonistic agents against various plant pathogens and exists in the rhizosphere soil of *P. notoginseng*, potentially plays a role in maintaining the microecological balance, as protective microbes, by suppressing soil-borne pathogens (Tagawa et al., 2010; Miao et al., 2016; Xiong et al., 2017). Similarly, *Epicoccum* and *Penicillium* spp. can produce numerous biologically active compounds that act as bacterial antagonists and plant growth promoters (Fávaro et al., 2012; Franke-Whittle et al., 2015). As potential pathogens, *Ilyonectria* spp. mainly caused root-rot disease (Farh et al., 2018). *Tetracladium* and *Leptodontidium* spp., which belong to the Leotiomycetes class, also were responded to soil sickness (Tan et al., 2017a). Some pathogenic fungi including *Ilyonectria*, *Tetracladium*, and *Leptodontidium* spp., as indicators of soil sickness (Xiao et al., 2016a; Tan et al., 2017a), markedly increased with increasing cultivation years (Figure 5B). The accumulation of allelochemicals (root exudates) from *P. ginseng* plants, such as phenolic compounds, had an important effect on the fungal community as the years of cultivation increased and could promote the growth of some pathogenic microorganism species (*Cylindrocarpon destructans*;Li et al., 2016; Zhang et al., 2017). Moreover, the increased abundance of pathogenic fungal genera (*Ilyonectria*, *Tetracladium*, and *Leptodontidium* spp.) in ginseng rhizosphere soil promoted the development of soil-borne diseases and increased death rates (Tan et al., 2017a; Dong et al., 2018). The decreased abundance of antagonistic fungi and increased abundance of pathogenic fungi could lead to an enrichment of pathotrophs, enhancing the rate of soil-borne disease with higher cultivation years (9-year-old ginseng plants). However, some fungal genera (*Ilyonectria*, *Tetracladium*, and *Leptodontidium* spp.) acting as indicators of soil sickness (Xiao et al., 2016a; Tan et al., 2017a) were not enriched, whereas some beneficial fungal genera (*Mortierella* spp.) were even enriched in the soil of ginseng plants with lower cultivation years (up to 5 years) in transplantation mode. This result revealed that the increase in cultivation years (up to 5 years) did not significantly influence the fungal community and function in the soil of ginseng plants in transplantation mode. Because of an accumulation of root exudates (even after transplantation), increased fungal pathogens were still observed with the 9-year-old transplanted ginsengs. Future studies including the effect of root exudates on soil microbial community are necessary to better characterize the alleviating mechanism of transplantation mode for *P. ginseng*.

### Relationship Between the Microbial Community and Soil Properties

The composition of soil bacterial and fungal communities was closely correlated with the soil properties (Figures 4, 5). The soil pH is a key determinant of the microbial community composition (Shen et al., 2013; Ren et al., 2018). Although ginseng plants grow better when cultivated in slightly acidic soils, a highly acidic soil could restrain ginseng growth by shaping the microbial community (You et al., 2015; Zhou et al., 2015). For instance, plant growth-promoting and yield-enhancing Bacilli, which were positively correlated with the soil pH, have been found in moderately acidic soil (Yadav et al., 2011; Kumar et al., 2012). The relative abundances of some fungal genera (*Mortierella* and *Epicoccum* spp.; which preferentially grow in slightly acidic soils) decreased, and other fungal genera (*Ilyonectria*, *Tetracladium*, and *Leptodontidium* spp.), preferring highly acidic soils, increased after the soil pH decreased, which could affect the growth of ginseng plants.

Microbes have been shown to be dominant drivers of biogeochemical cycles including nutrient cycling in soils (Wang et al., 2019b). Shifts in the relative abundances of some microbes with differing pH values can influence the nutrient availability in soils (Rubenecia et al., 2015; Tan et al., 2017b). Thermoleophilia class, a predominant Actinobacteria phylum subgroup that was negatively correlated with OM, is known to be involved in the degradation of OM in soils (Zhou et al., 2015). *Nitrospira* spp., belonging to Nitrospira class, was positively correlated with AN. These are nitrite-oxidizing bacteria that play pivotal roles in the N cycle (Pii et al., 2016). Betaproteobacteria class positively correlated with the soil pH and negatively correlated with OM levels have also been found by Zhou et al. (2015). However, our results showed that Alphaproteobacteria class was negatively correlated with AP and that Gammaproteobacteria class was negatively correlated with AP and OM; the opposite trend to that which has been previously reported (Zhou et al., 2015). Thus, Betaproteobacteria and Gammaproteobacteria classes may also be involved in the degradation of OM in soils. We also found that soil fungal communities were also closely correlated with soil properties (Figures 4B, 5B). Furthermore, *Mortierella* spp. were positively correlated with AP and has been suggested to be an important component of phosphorus cycling by Curlevski et al. (2010); in contrast, some potential genera of pathogens (*Ilyonectria*, *Tetracladium*, and *Leptodontidium* spp., among others) were positively correlated with the plant age and AK (Figures 4B, 5B). Our results indicated that low pH may inhibit the growth of some microbes involving nutrient element cycles and antifungal activity, leading to low AP or AN level and high abundances of some potential pathogens with an increasing age of *P. ginseng* plants.

## Conclusion

During prolonged growth (up to 9 years) of *P. ginseng* plants, the rhizosphere microbial diversity, abundance of some microbes and functional richness associated with nutrient element cycles and antifungal activity decreased, while the abundances of some potential pathotroph fungi increased. The abundance of some bacterial classes (Thermoleophilia, Bacilli, and Nitrospira) and fungal genera (*Mortierella*, *Epicoccum*, and *Penicillium* spp.) decreased with increasing age of *P. ginseng* plants. Conversely, the abundance of some fungal genera (*Ilyonectria*, *Tetracladium*, and *Leptodontidium* spp.) increased under the same conditions. Although microbial community diversity decreased, and the microbial composition and function changed with increasing number of years of cultivation, compared with cultivation for 2 years, cultivating ginseng plants for up to 5 years did not significantly influence the microbial communities of the ginseng rhizosphere soil in transplantation mode. Some fungal genera (*Ilyonectria*, *Tetracladium*, and *Leptodontidium* spp.) were not enriched in response to continuous cropping and some microbes (Thermoleophilia, Nitrospira, and *Mortierella* spp.) were enriched even in the soil of ginseng plants cultivated for up to 5 years in the transplantation mode.

## Data Availability Statement

Publicly available datasets were analyzed in this study. This data can be found here: sequences were deposited in the NCBI short read archive under accession numbers SRP131809 and SRP129584.

## Author Contributions

QW and YZ conceived and designed the study. QW, HS, and CX contributed to all experiments in this study. QW wrote the manuscript. QW and ML collected all the samples. ML helped with the data analysis of the Miseq sequencing. YZ helped to draft the manuscript. All authors contributed to the article and approved the submitted version.

### Conflict of Interest

The authors declare that the research was conducted in the absence of any commercial or financial relationships that could be construed as a potential conflict of interest.
